# Autologous platelet-rich fibrin promotes wound healing in cats

**DOI:** 10.3389/fvets.2023.1180447

**Published:** 2023-05-12

**Authors:** Anamika Changrani-Rastogi, Krutika Swadi, Mitali Barve, Niyati Bajekal

**Affiliations:** ^1^Dhirubhai Ambani International School, Mumbai, India; ^2^Private Veterinary Practice, Posh Vets Clinic, Mumbai, India

**Keywords:** cat, platelet therapy, autologous platelet-rich fibrin, cutaneous wound healing, regenerative medicine

## Abstract

Street cats commonly present large skin wounds that pose significant challenges in veterinary practice. Platelet-rich fibrin (PRF) is a second-generation platelet concentrate increasingly used in humans to promote wound healing. Ease of use and clinical success in humans has prompted interest in using PRF in veterinary practice. However, until now, there is no reported study on the use of autologous PRF in feline wound management. This study evaluated the effect of application of autologous PRF in cats with naturally occurring cutaneous wounds. 16 cats with full-thickness cutaneous acute/subacute wounds were randomly allocated to PRF or Control (standard care) groups. Each cat was enrolled for 2 weeks. PRF was prepared according to previously described procedures. PRF was applied on Days 1 and 4 in addition to standard wound care. Wound size was measured using tracing planimetry. Wound surface area was calculated using SketchAndCalc^™^ software on scanned tracing images. Average wound sizes at enrolment were 8.39 cm^2^ (Control) (standard deviation (SD) 5.08 cm^2^) and 9.18 cm^2^ (PRF) (SD 3.71 cm^2^) (range 2.42–15.97 cm^2^). By Day 14, the mean wound size for the Control group was 2.17 cm^2^ (SD 1.52 cm^2^) and for the PRF was 0.62 cm^2^ (SD 0.44 cm^2^) (*p* = 0.015). At Day 14, the PRF group showed mean 93.85% wound contraction with SD 3.66, while the control group showed mean 76.23% wound contraction with SD 5.30 (*p* = <0.0001). Based on the results, PRF could be further investigated to promote wound healing in cats as a low-risk and convenient adjunctive therapy.

## 1. Introduction

Street cats commonly suffer from large, infected wounds. Dog bites, territorial cat fight injuries, and even minor cuts may turn into open infected wounds with environmental contamination and oral bacteria (1). Cutaneous wounds in cats are generally slow to heal, even compared with dogs (2), and present considerable challenges in veterinary medicine as well as a significant economic burden (3). There is a need to develop cost-effective solutions to enhance wound healing in cats, particularly in countries with large stray cat populations.

Platelets are central to the healing of skin wounds. Growth factors provided by platelets play a key role in the proliferation phase of healing, including fibroplasia, reepithelialisation and neovascularization (4–8). Platelet-related products have been developed for use in modern regenerative medicine. Platelet-rich fibrin (PRF) is a second-generation platelet derivative that was first used in 2001 by Choukron et al. (9). PRF is a natural bioscaffold, providing a biophysical and biochemical milieu to promote tissue healing and regeneration. It contains platelets, leukocytes, cytokines and adhesive proteins such as fibrinogen, fibronectin, vitronectin, and thrombospondin-1 (10). PRF has increasingly been used in human medicine in the treatment of wounds from varied etiologies such as burns and diabetes (11–13). The evidence supporting its effectiveness is growing, with success *in vitro* and *in vivo* studies (14–18). This clinical success, in addition to other appealing properties of being autologous, and simple and inexpensive to produce, has led to an interest in using PRF in veterinary medicine (19, 20). Early, limited studies have examined autologous PRF use in donkeys (21) and dogs (22), with one preliminary study using heterologous canine-origin PRF in four cats with naturally occurring wounds (23).

To our knowledge, no studies have examined the use of autologous PRF in cutaneous wound management in felines. The purpose of our study was to evaluate the effectiveness of PRF as an adjunctive treatment in feline cutaneous wound management. Our hypothesis was that the use of autologous PRF will reduce the time for second intention cutaneous wound healing in cats. To test our hypothesis, we compared wound healing parameters between wounds treated with PRF and those undergoing a standard wound management protocol on full-thickness cutaneous wounds in cats.

## 2. Materials and methods

Cats were recruited at three locations in Mumbai, including a low-cost spay-neuter centre, a private veterinary clinic and a non-governmental animal shelter. Cats with one or more cutaneous wounds were screened to determine eligibility. Select photographs of cats taken by the rescuers are included in the Supplementary Material. The wounds had to be either acute (less than 1 week old) or subacute (1–2 weeks old), at least 2 cm^2^ in size, and of full thickness; the location and cause of the wound were not considered. A general physical examination was done by the attending veterinary doctor. If a wound was deemed suitable for surgical closure, it was excluded. For others, complete blood counts and serum biochemistry analyses were done. Cats with comorbidities and other medical conditions that could affect wound healing such as anaemia, hepatitis, kidney disease, malnutrition, and diabetes mellitus were excluded. Cats with white blood cell (WBC) counts up to 25,000/µL were considered as eligible if all other blood values were normal. For cats who fulfilled the inclusion criteria, rescuers provided informed consent. All eligible cats were enrolled with no rescuer refusing participation. The study was conducted at a private veterinary clinic, staffed with one veterinary surgeon and two veterinary doctors, all three of whom participated in the study. Each cat was enrolled in the study for 2 weeks with five clinical visits on Days 1, 4, 7, 10, and 14 at the study location. The cats were housed in a paid foster service for 2 weeks. Following the two-week study period, the rescuers were provided the option of continuing care at the vet and foster if needed. The enrolled cats were randomly allocated into PRF or Control groups using Microsoft Excel (RAND function).

### 2.1. Wound treatment protocol: PRF and control

All wounds were determined to be contaminated, based on gross examination noting edema and/or purulent exudate, and needed debridement. Debridement was done on Day 1 for all wounds using a surgical blade to remove necrotic tissue to create a fresh wound bed. At subsequent visits, no debridement was necessary. For the Control group, standard wound care dressing was done at each visit on Days 1, 4, 7, 10, and 14. Standard wound care included using non-adhesive Bactigras® dressing and Triple Antibiotic Ointment, with soft gauze dressing to collect excess discharge as needed. For the PRF group, the PRF application was done on Days 1 and 4 in addition to standard wound care on Days 1, 4, 7, 10, and 14. All cats received antibiotics (Clindamycin 15 mg/kg once a day orally for 7 days) and anti-inflammatory medication and pain relief treatment (Meloxicam at a starting dosage of 0.1 mg/kg once on the first day of treatment followed by a dosage of 0.05 mg/kg once a day for 2 additional days). Following the end of the study period of 2 weeks, wound care was done as needed according to the healing progression and considering the convenience of the rescuers for making clinic visits until wound closure was reached.

### 2.2. Platelet-rich fibrin preparation and application

**Figure 1:**
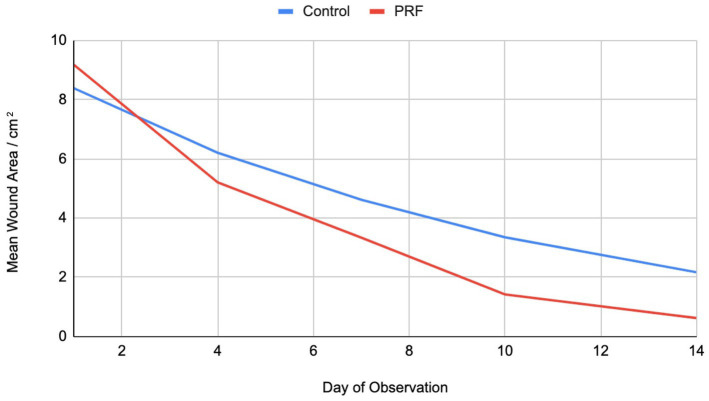
Mean wound area in cm^2^ over time.

**Figure 2:**
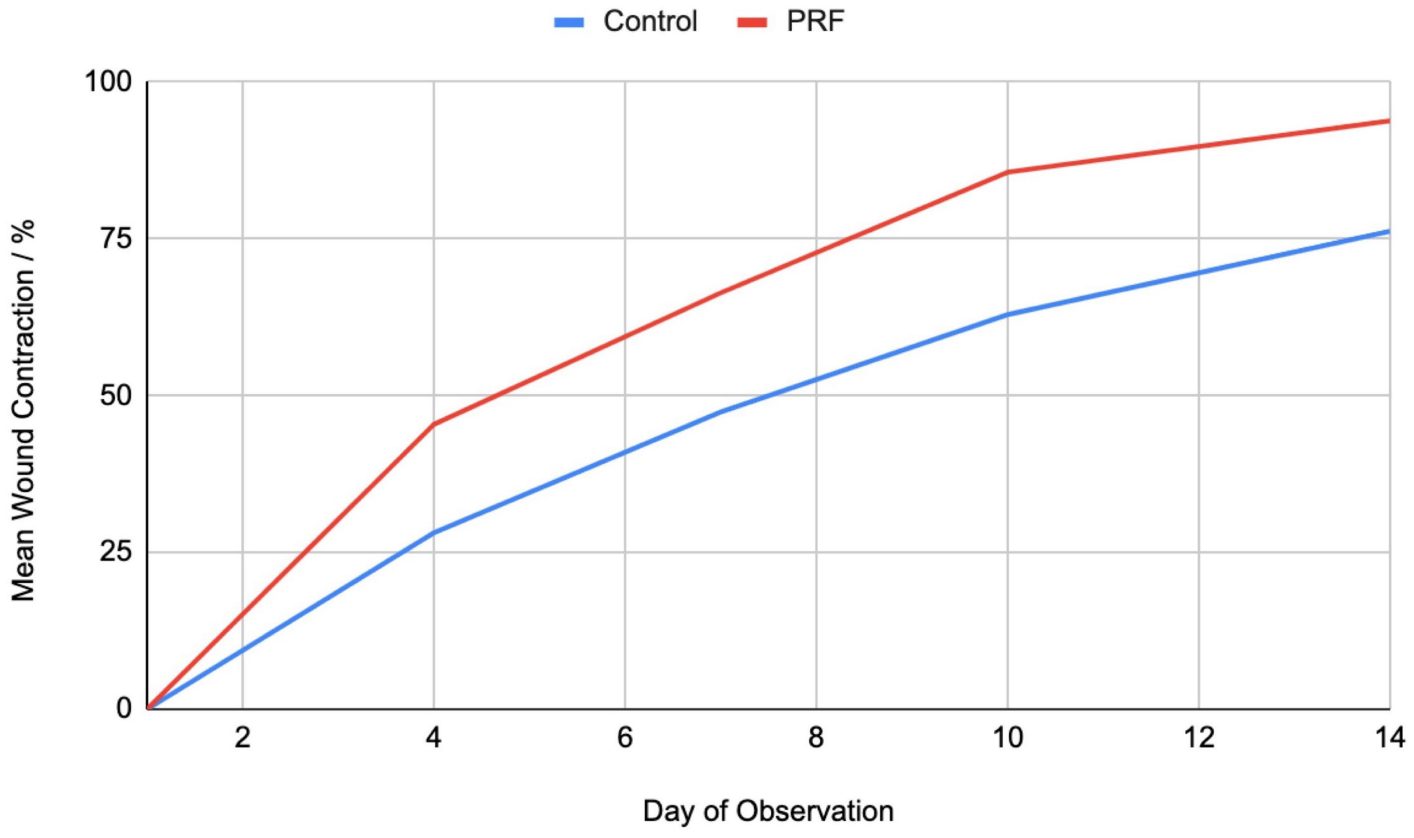
Mean % of wound contraction over time.

PRF clots were prepared as described in previous studies (9). Five mL of whole blood was collected from the jugular vein in a sterile 10 ml glass tube without any clot activators. Immediately after blood collection, it was centrifuged using Remi Clinical Centrifuge C-854/8 at 3500 rpm for 10 min at room temperature, which activated coagulation. The PRF clot formed between the red blood cell layer at the bottom and the plasma on the top. Using sterile tweezers, the PRF clot was removed and compressed between two slides. Using sterile surgical scissors, the PRF was separated from the blood clot. If the wound was large in size ~10cm^2^ or more, the PRF was cut into smaller pieces. The PRF or pieces of PRF were distributed across the wounds to ensure adequate coverage. Bandaging was done using sterile paraffin gauze to hold the PRF in place (Figures 1, 2).

### 2.3. Assessment of wound area

Tracing planimetry was done to document the wound size for all cats on Days 1, 4, 7, 10, and 14. This involved placing a double-layer tracing film on the wound and manually tracing the margin of the wound using a fine-point indelible marker (24). Tracings included the outer border of the wounds or the epithelial edge as the healing process progressed. The layer which had contact with the wound was disposed. Wound surface area was calculated by scanning the tracings and using SketchAndCalc^™^ software to assess the area of the scanned images (25). Wound depth was not considered. The wound area on the initial day of treatment was taken to be 100%. The change of wound size on Days 4, 7, 10, and 14 was compared with that of Day 1 and was reported as the percentage of wound contraction. The percentage of wound contraction (%WC) was calculated using the previously published formula: [(Wound Area on Initial Day − Wound Area on Day n)/Initial Wound Area] × 100 (26–29).

### 2.4. Statistical analysis

Means and standard deviations (SDs) were presented for linear variables. Means across groups were tested using the unpaired *t*-test. Statistically significant differences were assessed. Statistical analysis was done using STATA version 17 (StataCorp, United States).

## 3. Results

A total of 16 cats were enrolled in the study. All cats were male, ages unknown. The wounds were all infected with mean size at time of enrolment of 8.39 cm^2^ (Control) and 9.18 cm^2^ (PRF) (range 2.42–15.97 cm^2^, *p* = 0.72). At the end of the study period, a significant difference in the percentage of wound area reduction was noted between the two groups. By Day 14, the mean wound size for the Control group was 2.17 cm^2^ and for the PRF was 0.62 cm^2^ (*p* = 0.015). At Day 14, the PRF group showed mean 93.85% wound contraction with standard deviation of 3.66, while the control group showed mean 76.23% wound contraction with standard deviation of 5.30 (*p* = <0.0001) (Tables 1, 2).

**Table 1:** Wound area in cm^2^ over time.

Type	Statistics	Day 1	Day 4	Day 7	Day 10	Day 14
Control	Mean	8.39	6.21	4.62	3.35	2.17
	SD	5.08	4.16	3.10	2.40	1.52
PRF	Mean	9.18	5.21	3.34	1.42	0.62
	SD	3.71	2.44	1.82	0.72	0.44
Total	Mean	8.78	5.71	3.98	2.39	1.40
	SD	4.32	3.34	2.54	1.98	1.34
	*p* value	0.72	0.57	0.33	0.046	0.015

**Table 2:** Percentage contraction over time.

Type	Statistics	Day 4	Day 7	Day 10	Day 14
Control	Mean	28.15	47.39	62.95	76.23
	SD	5.94	6.63	7.47	5.3
PRF	Mean	45.46	66.41	85.66	93.85
	SD	7.98	9.36	4.05	3.66
Total	Mean	36.8	56.9	74.3	85.04
	SD	11.23	12.56	13.09	10.11
	*p* value	0.0002	0.0003	<0.0001	<0.0001

## 4. Discussion

PRF is a second-generation platelet concentrate with several advantages over the first-generation concentrate of Platelet-Rich Plasma (PRP). PRF is a three-dimensional fibrin matrix with supportive mechanical properties as well as platelets, growth factors and cells trapped in it that are released after a certain time. Unlike PRP, no additives are used, thereby avoiding any adverse reactions, and there is a higher concentration of immune cells with prolonged release of growth factors (GFs) (14, 30). The therapeutic effect of PRF and reduction in healing time may be explained by the aforementioned increase in concentrations of GFs, stem-like cells with high regenerative potential, hemostatic and antibiotic peptides, chemokines, in addition to the scaffolding provided by the fibrin matrix (31).

In felines in particular, PRF presents important advantages over PRP. Standard commercial centrifuge systems have not shown consistency in achieving ideal platelet concentrations in preparing feline PRP (32). Significant obstacles are presented by platelet aggregation. One study showed up to 40% of feline blood samples collected resulted in platelet clumping (32). Furthermore, felines are small blood volume animals, posing a challenge for PRP preparation which requires 12.5–15 mL of blood to produce 2–4 mL of PRP, whereas PRF preparation requires 4–5 ml of blood (33).

Additional benefits of PRF are the ease of preparation without the need for expensive equipment or advanced technical operator skills (9, 34–36). The methodology of PRF preparation is shown to be easily reproduced and consistent in the platelet concentration along with GFs, cytokines and hormones (37).

The limitations of the study are related to the intrinsic variability of PRF. PRF properties depend on a multitude of factors based on the characteristics of the patients and would thereby impact the responses to the treatment. A further limitation of employing PRF in wound management of cats is that autologous PRF is not possible when there are pre-existing hematological, metabolic or immune disorders with abnormal blood values. In our own prior experience, all cats with cutaneous wounds at our study sites routinely present with grossly infected, contaminated wounds. As such, we extended the allowable WBC count to 25,000/µL as the upper limit to be eligible for the present study (normal 20,000/µL).

Given that all the wounds were traumatic and contaminated, they were not uniform. In a study that artificially creates surgical wounds, identically sized control and treatment arms could exist in the same animal on different sides. However, given the large numbers of stray cats with naturally occurring traumatic cutaneous wounds at our study location, and the results observed in human use of PRF on wounds, it was considered ethically and scientifically sound to have a control group receiving standard care while studying the effects of PRF on naturally occurring wounds.

The PRF preparation in our study followed procedures as described in previous human and canine studies (9). We used 5 ml of autologous venous blood, considering the low-blood-volume nature of cats. PRF preparation from 4 to 5 mL of blood has been previously reported in human and canine studies (33, 38–40). Additionally, we used a glass tube in the PRF preparation. The use of a glass tube has been shown to activate coagulation during centrifugation, leading to the formation of a solid PRF matrix with entrapped platelets, leukocytes, proteins and GFs (41, 42). Further the PRF clot forms quicker and retracts easier in a glass tube as compared with a plastic tube (43).

Single use of platelet concentrates may not provide satisfactory clinical results, particular in infected wounds with high demands on healing processes (44). Repeated application of PRF has been employed in a previous study with heterologous canine PRF to manage wounds in compromised felines, with the intent to increase in-site concentration of cytokines and GFs (23). As such, we decided to have two applications of PRF per cat for our treatment protocol.

Wound area assessment was done using the tracing method, which is a reliable planimetry method (24, 45–47). This method was particularly suitable in our study with several wounds located on contoured surfaces, such as spanning the base of the ear to the back of the neck rendering single plane digital imaging unfeasible.

As this study was the first to examine the use of autologous PRF on wounded cats, there are several other aspects of PRF that merit investigation. For instance, this study did not consider street cats with comorbidities. However, since comorbidities are common among street cats, could PRF be used effectively even in such cases? Does the use of PRF lead to shorter duration for which medication is needed, including antibiotics and anti-inflammatory medicines? Which wound dressing will be most effective to use with PRF? Would a single application of PRF also promote healing? Such studies will contribute to the development of a reliable, reproducible and effective protocol to promote wound healing in cats using PRF.

## 5. Conclusion

This study demonstrated that autologous PRF can be used to significantly shorten the healing time in infected wounds in cats. Furthermore, this holds true for infected wounds with elevated WBC counts. Based on the results, this work supports further investigation into using PRF to promote healing in cats as a low-cost, low-risk and convenient adjunctive therapy.

## Data availability statement

The raw data supporting the conclusions of this article will be made available by the authors, without undue reservation.

## Ethics statement

Ethical review and approval was not required for the animal study because Platelet Rich Fibrin (PRF)’s safety has been established for over a decade for its wound healing properties. PRF has been effectively used in multiple medical specialties both in Human and Veterinary Medicine, including orthopaedics, dental and maxillofacial surgery, dermatology, ophthalmology, and cosmetic surgery. PRF is safe because it is autologous plasma with no additives. The current clinical study was carried out with cats brought in by their rescuers. All rescuers included in the study signed a written consent after having been explained all the relevant treatment and project information. The informed consent was discussed during the consultation and contained information about the treatment. All cats that participated in the study were directly overseen by a veterinarian to ensure no harm was incurred during study participation. Written informed consent was obtained from the owners for the participation of their animals in this study.

## Author contributions

AC-R and NB: conception and design of study, acquisition of data, and interpretation of data. AC-R: drafting the manuscript and revising. MB and KS: acquisition of data. All authors contributed to the article and approved the submitted version.

## Conflict of interest

The authors declare that the research was conducted in the absence of any commercial or financial relationships that could be construed as a potential conflict of interest.

## Publisher’s note

All claims expressed in this article are solely those of the authors and do not necessarily represent those of their affiliated organizations, or those of the publisher, the editors and the reviewers. Any product that may be evaluated in this article, or claim that may be made by its manufacturer, is not guaranteed or endorsed by the publisher.
