# Supplementary figures and images for: Autologous platelet-rich fibrin promotes wound healing in cats
Source: Front Vet Sci. 2023 May 12;10:1180447. doi: 10.3389/fvets.2023.1180447 (PMC10213361; doi:10.3389/fvets.2023.1180447)

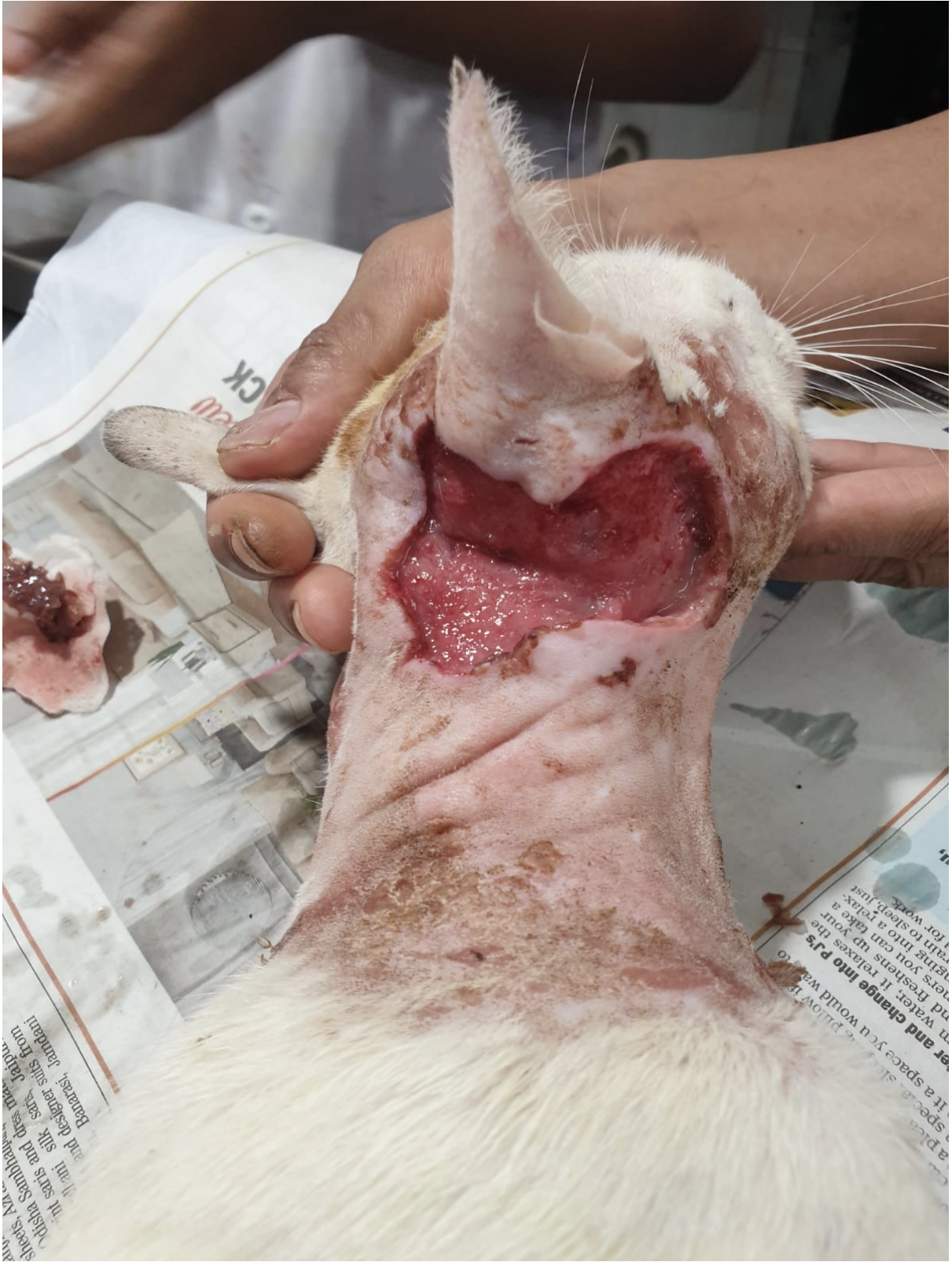

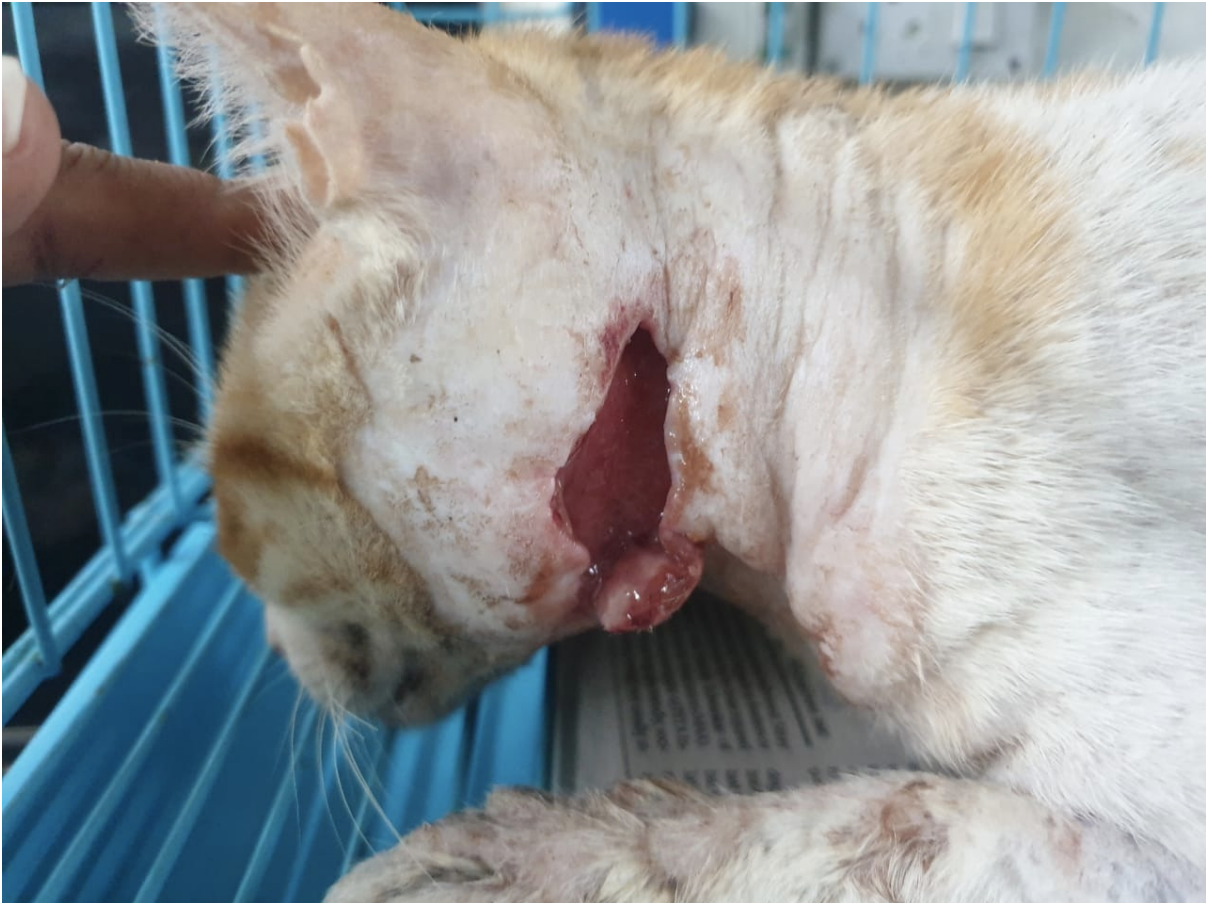

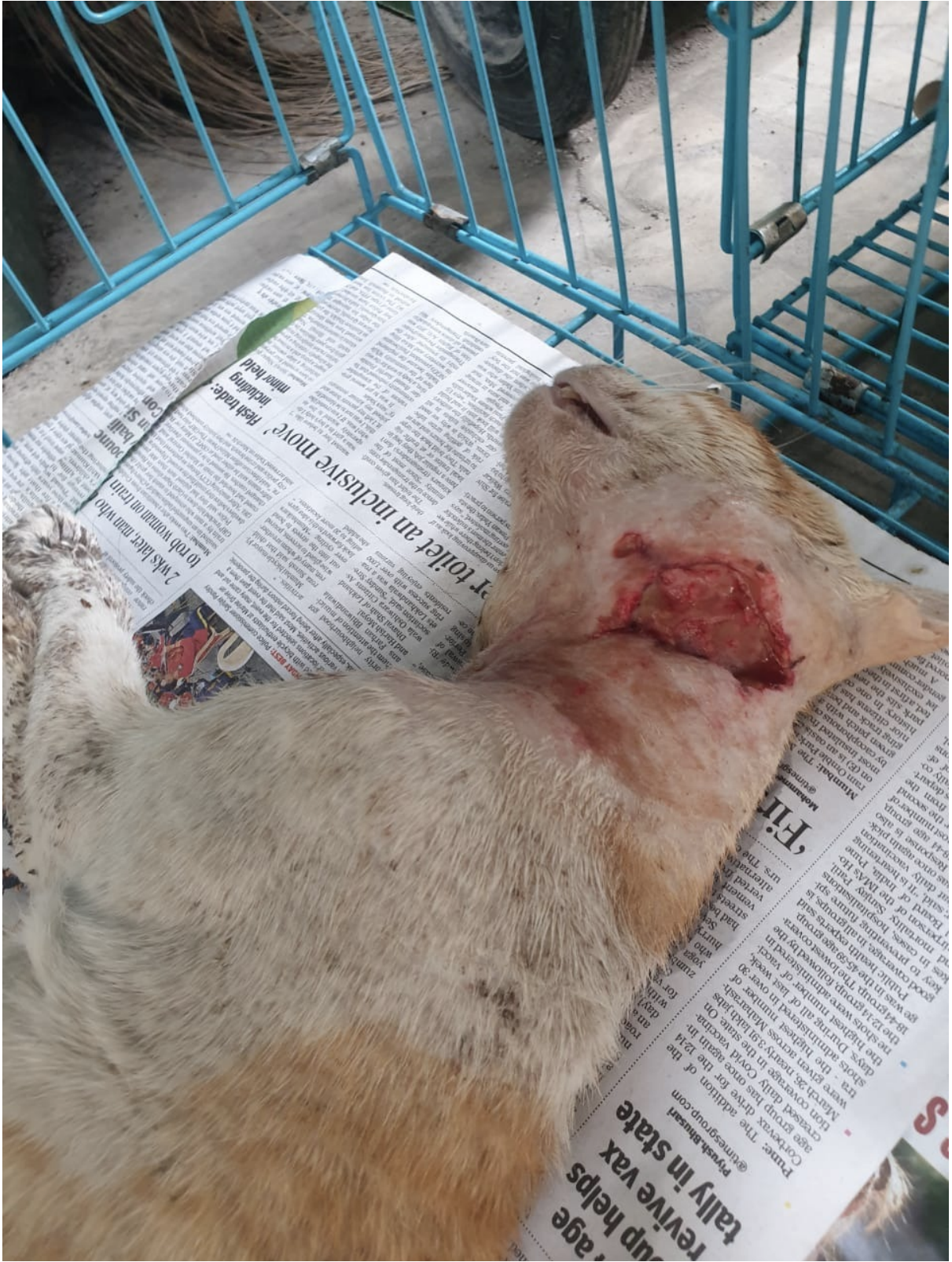

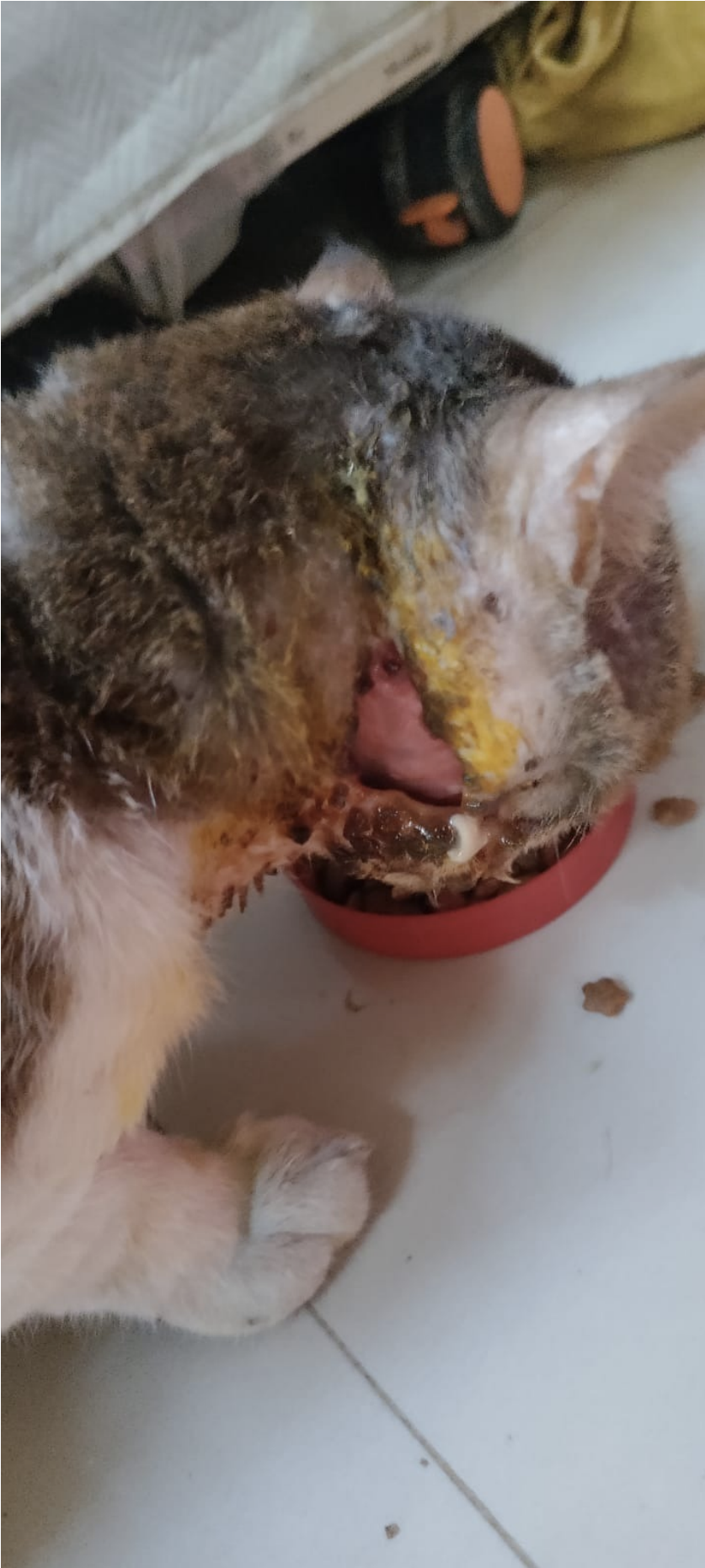

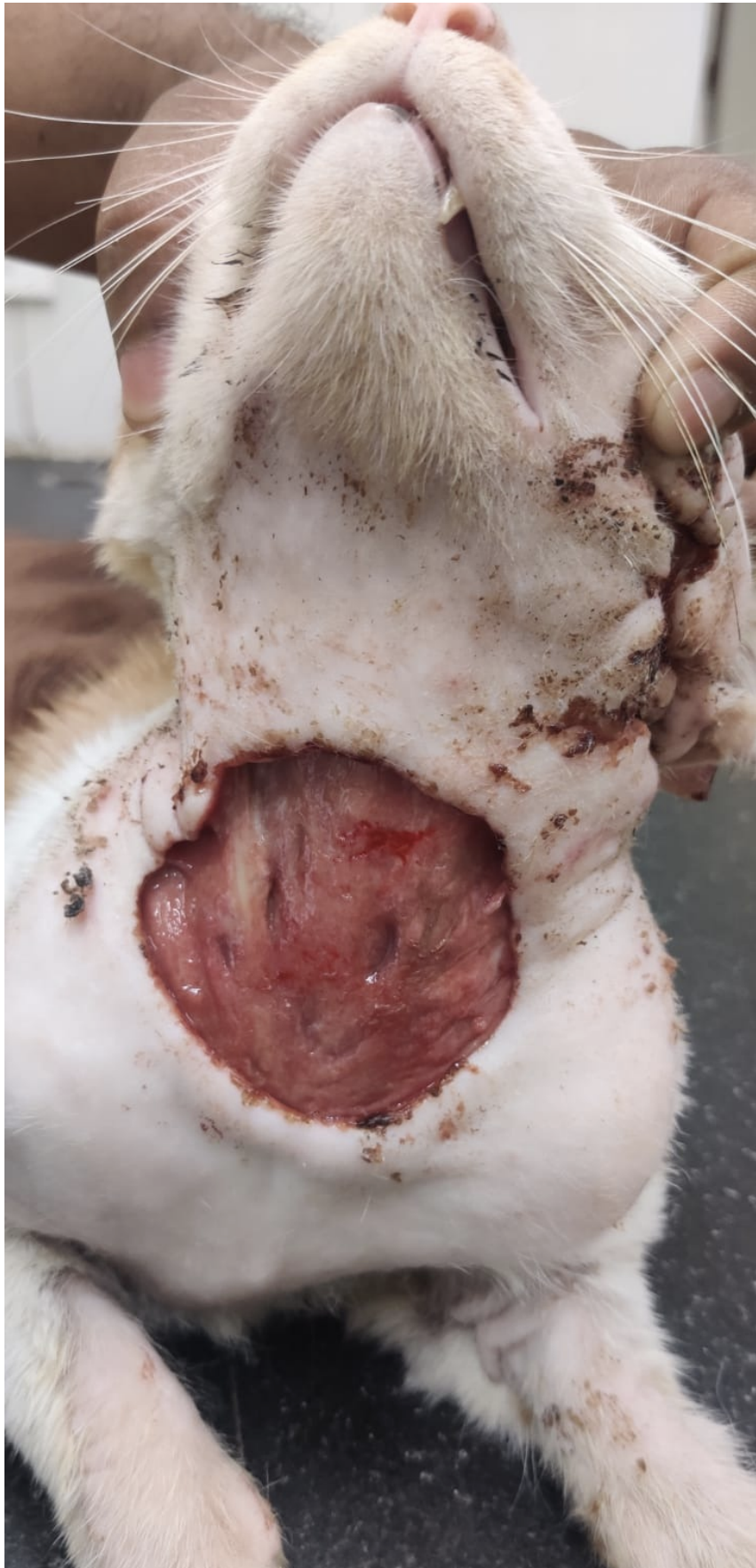

Supplement: Supplementary file 1 [file Data_Sheet_1.PDF]
